# Proton Beam Therapy for Multifocal Hepatocellular Carcinoma (HCC) Showing Complete Response in Pathological Anatomy After Liver Transplantation

**DOI:** 10.7759/cureus.25744

**Published:** 2022-06-08

**Authors:** Yinuo Li, Shosei Shimizu, Masashi Mizumoto, Takashi Iizumi, Haruko Numajiri, Hirokazu Makishima, Gong Li, Hideyuki Sakurai

**Affiliations:** 1 Department of Radiation Oncology, Graduate School of Comprehensive Human Sciences, University of Tsukuba, Tsukuba, JPN; 2 Department of Radiation Oncology, Proton Medical Research Center, University of Tsukuba Hospital, Tsukuba, JPN; 3 Department of Radiotherapy, Beijing Tsinghua Changgung Hospital, Beijing, CHN

**Keywords:** liver transplantation, pathological anatomy, radiation, proton beam therapy, liver cirrhosis, hepatocellular carcinoma

## Abstract

We describe a patient with multifocal recurrent hepatocellular carcinoma (HCC) who received proton beam therapy (PBT) and then underwent donation after brain dead (DBD) liver transplantation. The anatomy of the explanted diseased liver was examined pathologically post-transplantation. The patient was a 52-year-old male with hepatitis B virus infection and liver cirrhosis of Child-Pugh class B. Right lobe and caudate lobectomy were performed for primary HCC. However, three recurrent tumors appeared in the remnant liver in segments S2 (two sites) and S4, of sizes 23 mm, 10 mm, and 32 mm, respectively. Liver transplantation was required due to these multiple HCCs and liver cirrhosis, but the patient was ineligible for living donor liver transplantation (LDLT) based on Milan criteria. He was registered as a candidate on the waiting list for DBD transplantation. In consideration of the long waiting time for a deceased donor transplant for more than one year, the progression of multiple recurrent HCCs, and the risk of death, the patient had limited treatment options other than PBT for poor liver function and multifocal HCC and eventually received 65 GyE/18 fractions of PBT. Eleven months after the start of PBT, the tumors remained progression-free and liver function did not deteriorate, allowing the patient to wait for liver transplantation. After transplantation, the histopathology of the explanted liver showed that the left lobe of the liver treated by PBT showed no evidence of solid tumors and tumor cells in visual and microscopic examinations. There was also no significant damage to normal liver tissue. This case demonstrates that PBT is a prospective option for patients with HCC with poor liver function, multiple tumors, and no other treatment options. PBT can achieve control or even complete response of HCC while maintaining liver function and may be an effective pre-transplant method for tumor downstaging and prolonging survival. PBT may enable more people to wait for a donor liver or to become eligible for liver transplantation.

## Introduction

Primary liver cancer is the fifth most common cancer and the third most common cause of cancer-related mortality worldwide [1-2]. Among live cancer cases, 85-90% are hepatocellular carcinoma (HCC) [3]. About 80% of liver cancers are also attributed to chronic hepatitis B and C [4] since these diseases are important risk factors for cirrhosis, which has a strong tendency to proceed to HCC. Patients with HCC can be managed by surgical resection, transcatheter arterial chemoembolization (TACE), radiofrequency ablation (RFA), and liver transplantation [5-6]. However, surgery is only preferable for solitary early-stage HCC, and partial resection is not recommended for multiple and recurrent cirrhotic HCC [7]. Among non-surgical approaches, RFA gives good therapeutic outcomes for solitary HCC but not for multiple, large, and deeply located HCC [8]. Patients with cirrhosis leading to hepatic insufficiency should be carefully assessed for TACE because of the potential risk of liver failure or bleeding [9].

Liver transplantation is the optimal therapeutic option for liver cirrhosis and HCC [10], with excellent survival rates of >90% and >80% at one and five years, respectively [11]. Liver transplantation is mainly classified into living donor liver transplantation (LDLT) and donation after brain dead (DBD) liver transplantation [12]. In Japan and China, the Milan criteria are used to judge long-term eligibility for LDLT in patients with HCC [13-14]. However, the shortage of donor livers leads to long waiting periods for transplantation, and patients are in danger of death or tumor progression during this period, with a mortality rate of 11.3% [15]. Therefore, treating HCC during the pre-transplantation waiting period is necessary to reduce mortality and meet the criteria for transplantation.

Proton beam therapy (PBT) is an aggressive form of radiation therapy that offers advantages over photon-based radiation therapy through greater sparing of the normal liver surrounding the tumor due to its dosimetric properties based on the Bragg peak [16]. Thus, PBT reduces radiation-related hepatotoxicity while permitting escalation of the tumor dose [17]. At our center, we have shown the effectiveness of PBT for HCC, even in patients with cirrhosis and poor liver function, and we have found that PBT is tolerable and improves survival for patients with HCC with severe cirrhosis [18]. Here, we describe a case of multifocal HCC treated by PBT followed by DBD liver transplantation that showed a pathologically verified complete response to PBT with the disappearance of tumors.

## Case presentation

**Figure 1:**
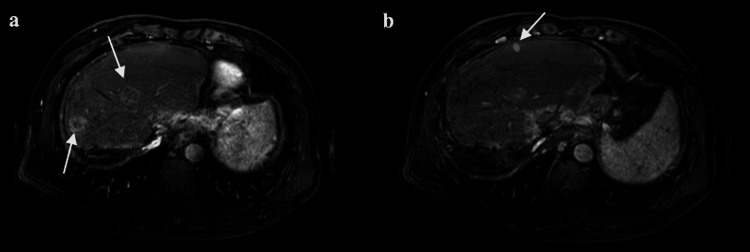
Arterial phase contrast-enhanced MRI revealed three hepatocellular carcinomas in the left lobe of the liver before proton beam therapy (a) Two high-intensity signal round-like nodules of 32 mm and 23 mm, respectively, in S4 and S2. (b) A 10-mm high-intensity signal round-like nodule in S2.

The patient was a 52-year-old male with chronic hepatitis B infection complicated by severe cirrhosis. At the initial onset of HCC, resection of the right lobe and caudate lobe of the liver was performed. Fifteen months later, a tumor recurred in the S3 segment, for which internal yttrium-90 (Y90) irradiation was performed. Since then, the patient had received targeted drug therapy with lenvatinib and regorafenib, but this treatment was not effective. One and a half years later, the tumors progressed and the patient was diagnosed with multiple HCCs by contrast-enhanced magnetic resonance imaging (MRI). The arterial phase (Figure 1) showed three high signal intensity nodules in the left lobe of the liver: two HCCs in S2 and S4 (Figure 1a), with sizes of 23 mm and 32 mm, respectively, and a round-like HCC located in S2 (Figure 1b) close to the hepatic peritoneum, with a size of 10 mm. In the portal venous phase, all three nodules had low signal intensity with a lack of contrast. The patient had severe cirrhosis and high hepatobiliary enzyme parameters of aspartate aminotransferase (AST) 54 IU/L, alanine transaminase (ALT) 58 IU/L, alkaline phosphatase (ALP) 158 IU/L, and gamma-glutamyl transpeptidase (γ-GTP) 99 IU/L before PBT.

The patient was listed as a candidate for DBD liver transplantation for multiple HCCs and severe cirrhosis. However, the long waiting time before transplantation placed the patient at risk of death due to tumor progression and poor liver function, and he was no longer able to receive surgery, chemotherapy with sorafenib, or stereotactic body radiation therapy (SBRT). Therefore, he was referred to our center. At the first visit, liver function was Child-Pugh class B, corresponding to Alb 3.9 g/dl, T-Bil 2.0 mg/dl, and PT% 79.7%. He had no hepatic encephalopathy and no abdominal dropsy with a small amount of ascites. The spleen was moderately enlarged, with the largest dimension of 14 cm. The patient had a very low platelet count of 50×109/L and suffered from esophageal varices, which indicated a high risk of rupture and bleeding.

**Figure 2:**
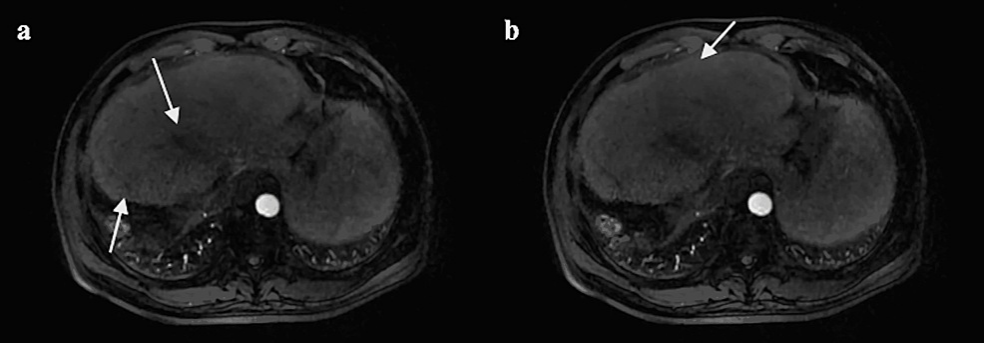
Arterial phase contrast-enhanced MRI nine months after proton beam therapy. (a) The tumor in S4 became invisible and one of the tumors in S2 became a low-intensity signal area. (b) The other tumor in S2 also disappeared.

A course of PBT was successfully performed and only grade 1 dermatitis was observed as acute radiation toxicity. Blood biochemical parameters related to liver function gradually decreased during PBT (Table 1). Nine months after the end of PBT, the tumor in S4 had disappeared on contrast-enhanced MRI (Figure 2a). The larger tumor in S2 showed a low-intensity internal signal area, and the smaller S2 tumor had disappeared (Figure 2). Liver structures remained intact without significant deformation, except for slight atrophy in the irradiated area of the tumor. Blood chemistry parameters before, during, and after PBT are shown in Table 1. Within 11 months after the end of PBT, the three tumors were completely controlled and the patient was in good condition to wait for liver transplantation, although cirrhosis was progressing very slowly. To date, after successful liver transplantation, there has still been no progression of tumors.

**Table 1:** Changes in blood chemistry before, during, and after PBT AST, aspartate aminotransferase; ALT, alanine aminotransferase; LDH, lactate dehydrogenase; ALP, alkaline phosphatase; γ-GTP, γ-glutamyl transpeptidase; T-Bil, total bilirubin; Alb, albumin; AFP, α-fetoprotein; PIVKAII, protein induced by vitamin K absence or antagonist.

Parameter	Before PBT	During PBT	3 months after PBT	8 months after PBT	10 months after PBT
AST (U/l)	54	26	52	29	74
ALT (U/l)	58	22	55	38	48
LDH (U/l)	201	154	222	/	/
ALP (U/l)	158	99	234	217	256
γ-GTP (U/l)	99	47	128	86	/
T-Bil (mg/dl)	2.0	1.0	0.94	1.1	2.7
Alb (g/dl)	3.9	3.5	3.5	4.0	3.5
AFP (ng/ml)	28	5.0	1.79	1.53	1.33
PIVKAII (AV/ml)	1322	86	50.26	12.15	16.53

Proton beam therapy

**Figure 3:**
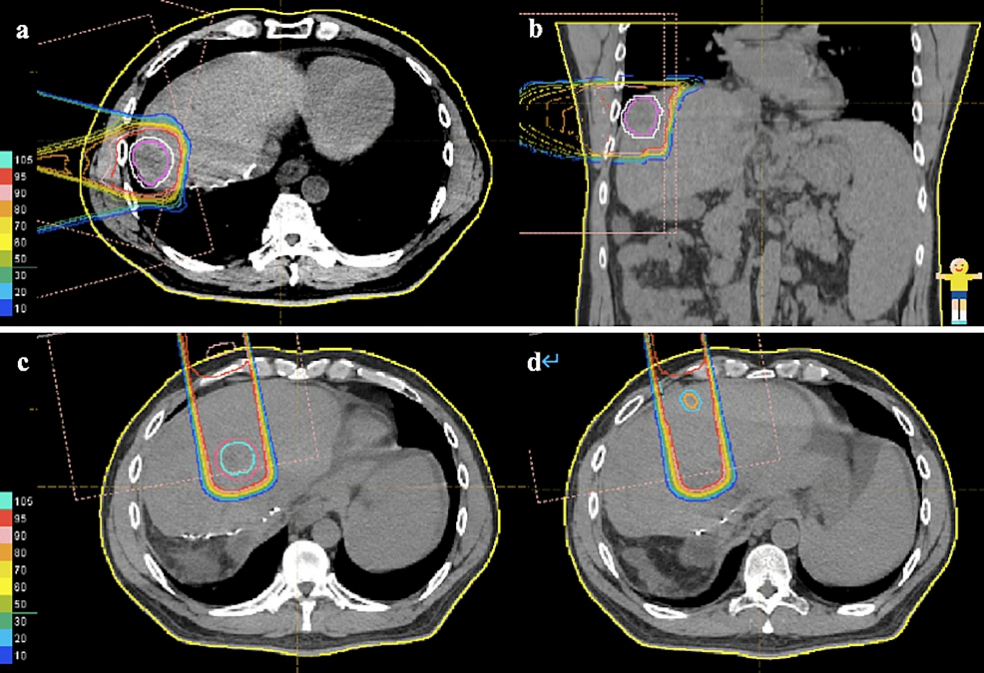
Dose distribution diagrams for proton beam therapy for HCC showing isodose curves ranging from 100% to 10% of the stated dose with 10% intervals shown as different colored lines Normal liver beyond the blue line was not irradiated. (a) Tumor in S4 in a cross-sectional view. (b) Tumor in S4 in a coronal view. (c, d) S2 segment tumors (two sites) in cross-sectional views.

Before proton therapy for HCC, our Proton Beam Therapy Center usually implants a metal target near the tumor to assist with localization. Laser-targeting was used during patient position setting, followed by fluoroscopy to match bone structure and correction, and finally adjustment around the position of the target. Respiratory synchronization sensors and 3D tumor motion-monitoring techniques were also used. The clinical target volume (CTV) was defined as the gross tumor volume (GTV) plus a margin of 5 to 10 mm in all directions. A margin of 5 mm was added to the tail axis to compensate for respiration-induced liver motion. A margin of 5 to 10 mm was added by expanding the multileaf collimator and adjusting the range shifter to cover the entire CTV, as shown in Figure 3.

The patient was administered once daily PBT for a total of 65 GyE/18 fractions. Initially, we performed 0-33 GyE/10 fractions of PBT, and the tumors were observed to shrink in size. Therefore, for the final 32 GyE/8 fractions, the radiation coverage was reduced to a good fit for the tumors and the spread-out Bragg peak (SOBP) was shortened. At the end of PBT, the patient had only Grade 1 dermatitis with PS 0 and no other acute phase reactions. The dose distribution diagrams (Figure 3) show that the tumor in S4 was irradiated lateral to two gates of the proton beam in cross-sectional and coronal views (Figure 3a and Figure 3b) while the two tumor lesions in S2 were irradiated with one gate of the proton beam obliquely from above (Figure 3c and Figure 3d).

Liver transplantation and pathological anatomy

The patient had one HCC >3 cm before PBT, which exceeded the Milan criteria (one lesion <5 cm; three lesions each <3 cm), and thus was not eligible for LDLT. However, he was registered as a candidate for DBD transplantation before PBT. At 11 months after the end of PBT, radiology showed no evidence of a tumor in the liver and no extrahepatic distant metastases or vascular invasion. The patient also had a good general condition. At this point, a donated liver became available and the patient underwent DBD liver transplantation in China. The pathological anatomy of the diseased liver showed that all three tumor lesions had been replaced by fibrous nodules. Two grayish-white nodular masses on the cut surface of the diseased liver were found. Among them, the size of the larger one was 8.5*5.5*6.5 cm, which was adjacent to the peritoneum of the liver and the hepatic cutaneous margin. About 2 cm away from the large nodule, the small nodule was observed, which was 3*4*5 cm. The liver section was uniformly solid and moderate in character. Microscopic findings revealed normal hepatocytes in the nodules had been replaced by hyperplastic fibrous tissue and reactive small bile ducts with high infiltration of lymphocytes and neutrophils. No cancer cells were seen microscopically, and the tissue in the radiation area surrounding the tumors had a normal structure and was only subtly damaged. The patient is currently in good health with normal liver function.

## Discussion

Liver transplantation is the best option for HCC because it allows the management of both the tumor and liver cirrhosis. The number of candidates for transplants for HCC has increased in recent years and this now accounts for 15-50% of all liver transplantations [19]. Over 60% of liver transplants are performed due to cirrhosis [20]. The Milan criteria are used to determine eligibility for LDLT worldwide, and among these criteria, the following conditions need to be met for eligibility: one lesion <5 cm or up to three lesions that are each <3 cm, no extrahepatic manifestations, and no evidence of gross vascular invasion [21]. In addition, Chinese and Japanese human organ transplant regulations indicate that LDLT must take place between individuals with consanguinity or clear family relationships. In contrast, DBD liver transplants are only available from voluntary private donations, and thus, it is unknown when a patient will receive a donor liver [22]. The huge gap between the demand for liver transplantations and the number of donors has led to significant morbidity and mortality of patients during the waiting period for transplantation [23]. A study in the United States showed that 51% of transplant candidates waited for more than one year for a liver transplant in 2018 while data from 2016 indicated that about 44% of candidates on the liver transplant list did not receive a transplant within three years and 11.3% died during the waiting period [15]. For patients with HCC, only 25% survived after six months if managed without any treatment [24]. Among patients with multiple HCCs who were unable to receive treatment such as surgical resection or TACE, Ho et al. reported a one-year survival rate of 13.9% and median survival of only 2.8 months [25]. In our case, the patient had waited for more than one year for a donor liver. Without intervention during this time, he had a serious mortality risk. Thus, alternative therapeutic modalities had to be considered to prolong survival.

Radiation therapy for HCC is not the first recommended treatment in clinical practice. This is mostly due to the low tolerance of the liver to radiation, which can lead to radiation-induced liver disease (RILD), even at low doses [26]. SBRT and other conventional photon modalities are constrained by the radiation dose permitted to avoid adjacent organ damage since most of the liver is irradiated during treatment [27]. In contrast, PBT has a unique dose distribution defined by the Bragg peak, allowing the sparing of normal tissues and focusing the dose on the tumor area [28]. Sumiya et al. showed that PBT can protect against and prevent deterioration of liver function through accurate targeting, based on the almost constant levels of liver/biliary enzymes and total bilirubin (T-Bil) related to liver function during PBT, and minimal direct damage to the normal liver even in cases with abnormal pre-treatment enzyme levels [29]. A review at the University of Tsukuba [18] showed that PBT has superior therapeutic performance and encompasses a wide range of indications for HCC, including large tumors, tumor thrombosis, and cases with poor liver function. The five-year local control rates of PBT for HCC are consistently >80%, regardless of the radiation protocols used [18].

In this case, the 52-year-old male patient had already received several treatment options, including surgery, chemotherapy, and internal irradiation due to HCC. As a consequence of severe cirrhosis and multiple recurrences of HCCs, these treatments could no longer be used to maintain liver function, so PBT seemed to be the only therapeutic option. After PBT, tumors were controlled, liver function did not deteriorate, and survival was extended [25], which allowed the patient to wait for a liver transplant safely. Thus, PBT in HCC cases not meeting the criteria for liver transplant may alleviate the tumor burden and downgrade the stage, and in turn, make the case consistent with the criteria [30]. Currently, the main methods for downstaging are local ablation therapy and TACE [31]. Chen et al. concluded that PBT for local management of HCC can be regarded as a bridging intervention for advanced aggressive HCC or as an option for downstaging of HCC to achieve criteria for transplantation, especially when other treatments are not available [27]. Thus, for patients not meeting transplant criteria, PBT may be considered a new approach for downstaging to enable more patients to become eligible for liver transplantation.

## Conclusions

Our case illustrates that proton beam therapy is a promising new option for HCC with poor liver function and multifocality, where other conventional treatments are not suitable. Proton beam therapy enables control of multiple HCCs, even to the point of complete tumor response, while preventing the deterioration of liver function and allowing for prolonged patient survival. Furthermore, these findings suggest that proton beam therapy is a prospective pre-transplant modality. It might downstage the tumor, extend patient survival, and further allow more patients to wait for a donor liver or become eligible for liver transplantation, leading to more patients having access to radical treatment for HCC.
